# Cloak and Dagger: Alternative Immune Evasion and Modulation Strategies of Poxviruses

**DOI:** 10.3390/v7082844

**Published:** 2015-08-21

**Authors:** Susanna R. Bidgood, Jason Mercer

**Affiliations:** Medical Research Council-Laboratory for Molecular Cell Biology, University College London, Gower Street, London WC1E 6BT, UK; E-Mail: s.bidgood@ucl.ac.uk

**Keywords:** virus entry, exocytosis, exosome, vaccinia virus, immune evasion

## Abstract

As all viruses rely on cellular factors throughout their replication cycle, to be successful they must evolve strategies to evade and/or manipulate the defence mechanisms employed by the host cell. In addition to their expression of a wide array of host modulatory factors, several recent studies have suggested that poxviruses may have evolved unique mechanisms to shunt or evade host detection. These potential mechanisms include mimicry of apoptotic bodies by mature virions (MVs), the use of viral sub-structures termed lateral bodies for the packaging and delivery of host modulators, and the formation of a second, “cloaked” form of infectious extracellular virus (EVs). Here we discuss these various strategies and how they may facilitate poxvirus immune evasion. Finally we propose a model for the exploitation of the cellular exosome pathway for the formation of EVs.

## 1. Introduction

Through millions of years of coevolution, viruses have devised numerous strategies to invade, hijack, and turn host cells into virus assembly factories. In turn, human cells have evolved diverse mechanisms to detect and combat these invading pathogens. Many of these are employed at cellular locations that allow for detection and deployment of defence mechanisms prior to the virus gaining a foothold and initiating its replication cycle. Given that all viruses must bind to the host cell surface, enter host cells through direct fusion or endocytosis, and ultimately transit the host cytoplasm [1]; it is perhaps no surprise that cellular antiviral factors can be found on the cell surface, within endosomes, and in the host cytoplasm [2,3].

For instance, Toll-like receptors (TLRs), a family of pattern recognition receptors that detect repetitive or conserved pathogen structures are exclusively located at the cell surface and in endosomal membranes [2,4]. As TLRs recognise a broad range of pathogen associated molecular patterns (PAMPs), they are ideally located to detect and initiate an inflammatory response to invading viruses during cell entry [5]. Cells also express danger associated molecular pattern receptors (DAMPRs). These receptors detect host proteins aberrantly located due to damage or infection. For example, it was recently shown that C3 complement proteins bound to the capsid of non-enveloped viruses are detected by a DAMPR after cytoplasmic delivery [6]. This results in intracellular virus neutralisation, triggers mitochondrial antiviral signalling, and initiates proinflammatory cytokine secretion.

Cytoplasmic nucleic acid receptors detect viral genomes released into the cytoplasm. RIG-like Receptors sense foreign RNA, while cytosolic DNA is detected by a range of sensors including the AIM2-like receptors, and the recently identified cyclic GMP-AMP synthase [7,8,9,10,11]. In response to this multi-level defence, viruses have evolved different strategies to evade or disable these antiviral detection systems [12]. For most viruses this exclusively involves expression of immunomodulatory factors during the initial stages of infection that counteract the antiviral signalling triggered during entry.

Exceptions to this are the large DNA viruses such as members of the *Herpesviridae* and *Poxviridae* families [13]. In addition to immediate early expression of a subset of potent immunomodulators, these viruses package immune modulating proteins during assembly. Upon infection, these immune modulating proteins are delivered into the cytoplasm of the host cell to combat the intrinsic immune response before viral gene expression ensues [14,15,16,17].

Amongst the large DNA virus families, the poxviruses encode the greatest number of immune antagonising viral proteins. They dedicate 30%–50% of their ~200 genes to encoding immunomodulating proteins and thus display the most diverse range of immune evasion strategies [18]. The poxvirus family includes, variola virus the causative agent of smallpox, monkeypox virus, and vaccinia virus (VACV) [19]. Best known for its use as the vaccine during the global eradication of smallpox [20], today VACV serves as the laboratory model poxvirus.

Like all poxviruses, VACV is a large, enveloped double-stranded DNA virus, which replicates exclusively in the host cell cytoplasm [13]. Poxviruses are unique in that during replication they produce two forms of infectious particles: mature virions (MVs) and extracellular virions (EVs). Structurally, MVs consist of a biconcave core containing the viral genome and flanked by two proteinaceous “lateral bodies” (LBs). This is further surrounded by a single lipid bilayer viral membrane [21,22]. EVs consist of a MV-like virion surrounded by an additional cell-derived membrane containing cellular proteins and seven virus proteins not found in MVs [23,24].

During infection, MVs and EVs serve different purposes; the MVs are released from cells after lysis, and due to their exceptional stability are thought to be required for host-to-host transmission [13,25]. EVs, on the other hand, are released into body fluids where they are responsible for the dissemination of virions within tissues and between organs [26]. As such, the outer EV membrane is thought to help virions evade immune detection while in circulation.

Thus, with a multitude of encoded immunomodulatory genes, the ability to package and deliver a subset of these directly into host cells, and two infectious virus forms that display different membranes containing divergent lipid and protein constituents, poxviruses pose a unique multifaceted challenge to the host immune system. As Smith *et al.* recently presented an extensive review of the poxvirus immunomodulatory proteins, which are expressed during infection [18], here we will review and discuss the intrinsic means of immune evasion “cloak” and immunomodulation “dagger” exhibited by poxviruses. In particular, we discuss three strategies used by the *Poxviridae*: MV viral apoptotic mimicry and its potential role in immune suppression, the use of LBs as immune modulatory delivery packets, and membrane cloaking as a means to facilitate spread of EVs.

## 2. VACV Replication Cycle

**Figure 1:**
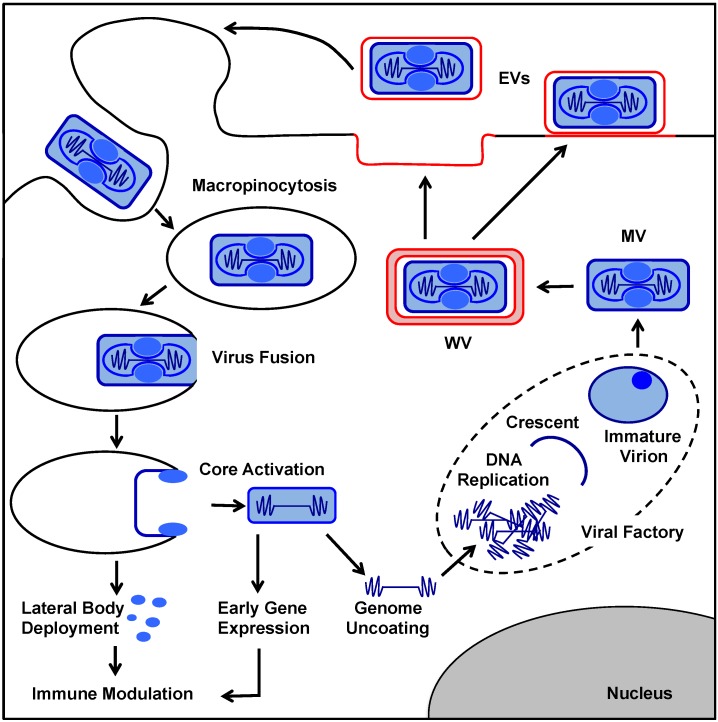
Vaccinia virus (VACV) replication cycle. The lifecycle of VACV begins when either, a single-membrane mature virion (MV) or double-membrane extracellular virus (EV), containing the genome, lateral bodies (LBs), and early transcription machinery, enters the host cell by inducing their own macropinocytic uptake. Upon fusion of the viral and the cellular limiting membrane of the macropinosome, the LBs dissociate from the core and are deposited in the cytoplasm. The LBs disperse, releasing virus host modulatory factors. Cores undergo activation concomitant with the initiation of early gene expression. Approximately one half of the proteins encoded by early genes serve an immunomodulatory function, while the remainder are required for genome uncoating and subsequent genome replication. Genome replication occurs in cytoplasmic viral factories where MVs are also assembled. Assembly is a highly complex multi-step process involving the formation of several non-infectious virus intermediates (crescents/immature virions). Once formed, MVs either exit cells by lysis or become wrapped by two additional cell derived membranes (red) which direct their exocytosis and thereby formation of EVs.

The lifecycle of poxviruses, illustrated in Figure 1, is a complex multi-step process beginning with the binding and internalisation of virions into host cells. Virus internalisation is followed by uncoating of viral genomes, and their subsequent amplification. In the final stages of the lifecycle, new virions are assembled and exit the cell to spread infection. Both VACV MVs and EVs enter host cells by inducing their own macropinocytic uptake [27,28,29,30,31]. This cellular endocytosis mechanism constitutes a transient, growth factor-induced, actin dependent process that leads to the uptake of extracellular fluid into large cytoplasmic vacuoles [27,32,33] Under non-pathological conditions macropinocytosis is used by cells for immune surveillance, clearance of apoptotic debris, and uptake of nutrients [34]. MV macropinocytic entry is triggered by phosphatidylserine (PS) in the viral membrane [27]. As the clearance of apoptotic debris is also dependent on PS exposure, it was proposed that VACV MVs use apoptotic mimicry as a means of inducing their cellular uptake. For EVs, the cellular binding factors, endocytosis receptors, and viral proteins that mediate these processes remain to be determined. It has however been demonstrated that the PS-bridging molecule Gas6 can boost EV infection in a PS-receptor dependent fashion suggesting that like MVs, perhaps EV entry involves apoptotic mimicry [35,36].

Fusion of both MVs and EVs from macropinosomes depends on the entry fusion complex (EFC), a macromolecular assembly of 12 viral proteins located in the MV membrane [37]. In addition, fusion of both virus forms is low pH dependent [15,38,39,40] indicating a requirement for macropinosome maturation during viral entry [41]. For MVs, acidification is required for removal of the EFC regulatory proteins A25 and A26 and subsequent EFC fusion activity [42]. Interestingly the MV-like particles that become EVs do not carry these negative regulators [23]. Instead for EVs, macropinosome acidification serves to disrupt the outermost EV membrane thereby exposing the underlying EFC to allow for fusion [23,28,39].

Upon fusion the lifecycle of VACV MVs and EVs converge, the viral cores containing the viral DNA, viral transcription factors, and RNA polymerases pre-bound to early promoters [43], are released into the host cell cytoplasm. The cores immediately undergo dramatic morphological changes switching from biconcave to oval; this process is termed activation. Core activation is marked by the uncoupling of the LBs, which remain behind with the fused viral membrane, the reduction of core proteins, the expansion of the core structure, and the initiation of early viral gene expression [15,44]. Activation does not require viral early gene expression suggesting that the process is intrinsically built into newly assembled particles [15,45,46].

The ~80 early genes are transcribed within cytoplasmic cores, then extruded and translated on host ribosomes. This gives rise to a set of early viral proteins required for DNA replication, intermediate gene transcription, and a wide array of immune modulation activities [13]. Amongst these early gene products is the viral AAA+ ATPase D5, which facilitates genome uncoating in collaboration with host ubiquitin, and proteasome activity [47,48]. Once released, the genome is replicated giving rise to large cytoplasmic viral factories. Intermediate and late gene expression occurs only from replicated genomes, resulting in the production of structural proteins and enzymes required for virion morphogenesis and proteins destined to be packaged into the newly assembled virions.

The formation of new infectious MVs requires no less than 40 virus encoded structural proteins and 10 virus encoded enzymes [49]. As recently reviewed by Liu *et al.*, the process begins with the formation of single crescent shaped membrane sheets and culminates with the formation of the characteristic brick shaped MV, having gone through a handful of distinct assembly intermediates [49,50]. Newly assembled MVs leave the cell by lysis approximately 72 hours after initial infection.

To overcome this relatively slow infection kinetic, a subset of the newly assembled MVs go on to become EVs, the second infectious virus form [23]. To speed the process of virus spread, the first round of EVs is released from cells as early as 6 hours after initial infection and their spread is enhanced by a novel form of superinfection exclusion, termed of superinfection repulsion [51]. For EV formation, the MVs acquire two additional membranes thought to be derived from virus-modified trans Golgi or endosomal membranes [52,53,54,55,56]. Once formed, these triple-membrane bound wrapped virions (WVs) are transported along microtubules to the cell surface, where they exit the cell by exocytosis [24,57,58,59]. Fusion of the outermost WV membrane with the plasma membrane results in the formation of cell surface bound double-membrane EVs. A subset of EVs are released from the cell surface, while others induce the formation of actin tails which propel the EVs away from the producer cell to facilitate cell-to-cell spread [57].

The complexity of the poxvirus replication cycle offers the infected cell a plethora of opportunities to target and neutralise infection. To combat this, the poxviruses dedicate nearly half of their genomic capacity to encoding cell and immune modulatory factors. Yet between the time of VACV exit from one cell and the initiation of early gene expression in the next, host modulatory factors are not being synthesised and thus the virions are potentially vulnerable to immune detection and destruction. To this end, the poxviruses MVs and EVs have developed several protein expression independent strategies to combat and evade host immune responses during virus entry and spread.

## 3. Immune Suppression during MV Entry

For host cell entry VACV MVs use an apoptotic mimicry strategy to trigger their macropinocytic uptake [27,60]. For this, the virus mimics an apoptotic cell or body by concentrating PS within its membrane in order to facilitate infection. Interestingly, apoptotic clearance is intimately linked with a dampening of inflammatory responses [61,62,63]. Engagement of the PS bridging molecules Gas6 or Protein S, with PS receptors Tyro3, Axl, or Mer (TAM receptors), has been shown to initiate enhanced transcription of TLR and cytokine suppressors SOCS1 and SOCS3 [64]. While initially hypothesised as a potential viral immune evasion strategy in 2003 [65], not until recently has the potential of viral apoptotic mimicry to serve for immune modulation come to light. A recent study by Bhattacharyya *et al.*, showed that PS-containing enveloped viruses complexed with PS-bridging molecules act as “super agonists” that activate TAM receptors to disable host immune responses [66].

For VACV MVs, envelope PS serves to trigger the signalling cascade (Rac1/Pak1/PI3K/PKC) needed for macropinocytosis [27,67,68]. While the receptors for both MVs and EVs remain elusive, the PS receptor Axl has been implicated in MV entry. For MVs, ligand-based receptor capture technology showed that VACV MVs on the cell surface bound a subset of six receptors, including Axl. Subsequent RNAi-mediated depletion of Axl was shown to reduce infection [69]. Interestingly, it has been suggested that VACV EVs may also use apoptotic mimicry for entry. Although whether EVs display PS on their outer envelope has not been investigated, both the PS-bridging molecule Gas6 and Axl overexpression were found to enhance infection [36].

Although no direct link between VACV apoptotic mimicry and immune modulation has been established, *in vivo* VACV infections result in the induction of anti-inflammatory cytokines including TGF-β and IL-10, prevent macrophage infiltration, and inhibit T cell maturation [70,71]. These processes are identical to those triggered during apoptotic cell clearance to dampen unwanted inflammatory responses. While this early immune suppression by VACV was proposed to be connected to unchecked replication, it is possible that this is rather due to engagement of PS receptors during the entry process.

## 4. Post Entry VACV Immunomodulation

Upon their cytoplasmic arrival viruses encounter a new subset of host defence mechanisms in the form of innate immune sensors [2,72]. These include factors that serve to detect and destroy the incoming viral capsids and genomes [2,3], as well as signalling proteins (PAMP receptors and TLRs) that may have been triggered during virus binding or endocytosis [73].

To overcome these innate defence mechanisms poxviruses bring their own subset of intrinsic immune modulatory proteins. The factors are packaged into the virus during assembly and reside in the two LBs found between the viral core and membrane. These enigmatic structures were first visualised by electron microscopy (EM) in 1956 [74]. As early as the 1960s, EM studies showed that LBs detach from VACV cores during the membrane fusion step of virus entry [44]. Biochemistry-based analysis of VACV MVs in the 1980s indicated that LBs were proteinaceous and that they were structurally distinct from both the viral core and membrane [75].

A function of poxvirus LBs was recently elucidated through investigation of VACV core activation. Using a variety of biochemical and imaging techniques Schmidt and Bleck *et al.*, demonstrated that one function of LBs is to serve as viral immunomodulatory delivery packets [15]. They identified three VACV proteins that reside in LBs, the phosphoprotein F17, the dual-specificity phosphatase H1 and the viral oxidoreductase G4 [15]. F17 is the third most abundant protein packaged into virus particles and accounts for approximately 69% of the LB proteinaceous mass [76]. While highly disulphide linked within virions, deposition of LBs into the reducing environment of the cytoplasm results in reduction of F17 and its subsequent proteasome dependent degradation. These findings led to the suggestion that F17 serves as the LB structural protein. In support of this, proteasomal degradation of F17 was found to be required for release of the LB resident protein, H1 phosphatase and its subsequent immunomodulatory activity [15].

**Figure 2:**
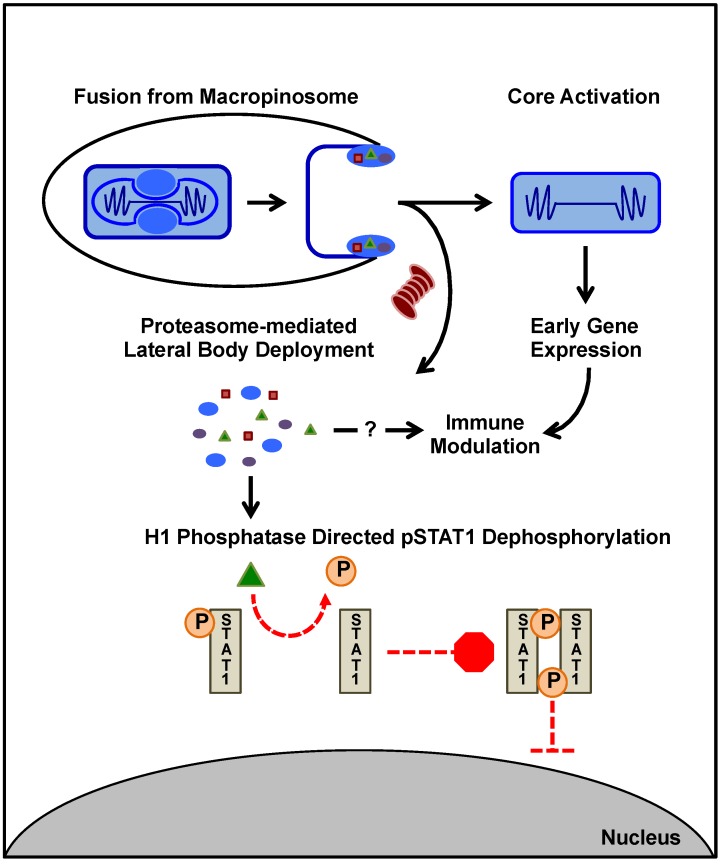
VACV LBs as Immunomodulatory Delivery Packets. After internalisation via macropinocytosis VACV particles undergo fusion with the limiting membrane of the macropinosome releasing the viral core into the cytoplasm. The released viral cores are “activated” as indicated by morphological changes and the initiation of early gene expression from within. Upon fusion, the LBs detach from the core and remain associated with the viral membrane. Once exposed to the cytoplasm, LBs are rapidly disassembled, with the major LB structural protein, F17, undergoing proteasome dependent degradation. Disassembly of the LB appears to facilitate release of other LB proteins and, in the case of the viral dual specificity phosphatase H1, is required for their action. Release of H1 from LBs, serves to shunt cellular antiviral transcription prior to the expression of early viral genes. To do this, H1 dephosphorylates phospho-STAT1 preventing its homodimerisation and nuclear translocation. To date only three LB components F17, H1, and a viral disulfide oxidoreductase G4 have been identified.

To date the viral phosphatase H1 is the only LB component with a defined role in immunomodulation. In response to viral infection, interferon-γ (IFNγ) induces the phosphorylation of the transcription factor STAT1 leading to its homodimerisation, nuclear translocation and subsequent induction of antiviral gene transcription [77]. LB-mediated delivery of H1 counteracts this antiviral response by dephosphorylating STAT1 to prevent its nuclear translocation and thereby block IFNγ-induced immune signalling [15,78] (Figure 2).

Currently, no immune modulatory roles for G4 or F17 have been identified. F17 is packaged at 27,000 copies per virion and is known to carry two proline-directed phosphorylation sites that can be phosphorylated by ERK1/JNK1/cdk1/cyclin B *in vitro* [79,80]. While mutation of these sites does not impact the assembly of virions, those that package a mutant form of F17 lacking these phosphorylation sites display defects in early viral gene expression [80]. Interestingly, post entry activation of mitogen-activated protein kinase (MAPK) signalling has been reported to be required for VACV early gene expression and genome replication [81]. Given the vast amount of F17 delivered by each virus during entry, it is tempting to speculate that these F17 phospho-sites may play an important role in modulating the cellular immune response initiated through MAPK signalling pathways [80].

The three identified LB proteins are expressed during the late stages of the viral lifecycle and packaged into assembling virions [49]. Together they account for ~70% of the LB mass with H1 and G4 each contributing to around 1% [15]. In addition to their LB residence, each is known to play an active role in the viral life cycle. Both F17 and G4 are essential for viral morphogenesis [80,82], and H1 for assuring the transcriptional competence of newly assembled virions [83]. As testament to the importance of LBs, all poxviruses identified to date carry them [15]. Thus, it will be of major interesting to determine if the factors that make up the remaining LB mass also play multiple roles during the virus lifecycle; perhaps facilitating viral replication or assembly in addition to modulating host immune defences.

## 5. VACV EV Formation

### 5.1. Overview: From MV to EV

The production of a double-membrane bound second infectious virus form, EVs, is entirely unique to the *Poxviridae* family. As the outermost EV membrane is unstable these particles are not thought to be particularly effective for transmission between hosts [39,84,85]. Instead, evidence suggests that the virus evolved this strategy as a way to cloak MVs during the spread of virus within and between host tissues.

**Figure 3:**
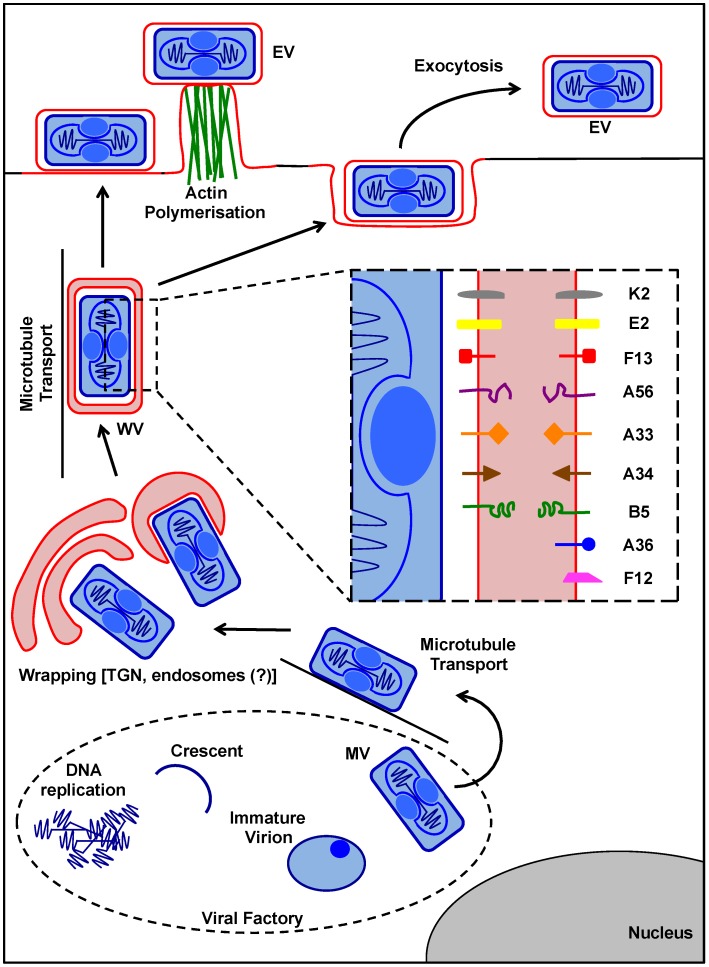
VACV MV to EV. After assembly within the cytoplasmic virus factory, a subset of MVs are transported on microtubules to the site of wrapping in the region of the microtubule organising center. The wrapping membranes are thought to be derived from the trans-Golgi network (TGN), or potentially LEs, and EEs after recycling of EV proteins from the plasma membrane. MV wrapping results in the formation of a triple membrane WV. WVs contain 9 additional proteins (illustrated in the zoom box) not found in MVs that direct the wrapping, post-wrapping transport, exocytosis and virus spread. Once formed, WVs are transported to the cell surface on microtubules. Upon reaching the plasma membrane the outermost WV membrane undergoes fusion thereby exocytosing the underlying double-membrane enveloped EV. EVs can either remain associated with the producer cell, detach from the cell surface to mediate long distance spread, or by the action of actin tails be propelled away from the cell surface to facilitate cell-to-cell spread. VACV: vaccinia virus; MV: mature virion; WV: wrapped virion; EV: extracellular virion; LE: late endosome; EE: early endosome.

The formation of VACV EVs is a highly orchestrated, multi-step process involving intracellular virion transport, membrane wrapping, and exocytosis events outlined in Figure 3. The MVs destined to become EVs are actively transported along microtubules away from the viral factories towards the microtubule organising centre, the site of wrapping [86,87]. Of note, the MVs that become EVs do not carry the viral fusion regulatory proteins A25 and A26 [23] suggesting that the wrapping of an individual MV is pre-determined during morphogenesis. During wrapping MVs are enveloped by a double cell-derived membrane to become WVs containing three membranes. These additional membranes contain a set of viral proteins not found in MVs: A33 [88], A34 [89], A36 [90], A56 [91], F12 [92], F13 [93], B5 [94], E2 [95] and K2 [96,97]. These proteins are involved in MV wrapping (B5 and F13), WV transport (A36, E2, and F12), actin tail formation (A33, A34, and A36), and EV superinfection exclusion (A56 and K2). Once transported to the cell periphery, the outer WV membrane fuses with the plasma membrane resulting in the release of double-membrane EVs. The majority of EVs remain cell-associated; however a subset initiate the formation of actin tails, which drive the EVs away from the producer cell, while others entirely detach from the cell surface to mediate long distance dissemination [57].

### 5.2. WV Formation

How MVs are wrapped by two additional membranes to become WVs is not fully understood. Early attempts at identifying the cellular source of the double-membrane that envelopes MVs established that brefeldin A abrogates the production of EVs without impacting MV production. As brefeldin A inhibits the formation of COPI vesicles thereby resulting in the collapse of the Golgi into the endoplasmic reticulum, it was concluded that the Golgi or a post-Golgi compartment was involved in MV wrapping [98].

EM studies support the involvement of the trans-Golgi network (TGN), as WV membranes contain glycoprotein and glycolipid sugars which are only added in the late TGN [52]. When EV proteins are individually expressed, A56, B5 and F13 are found within TGN membranes and it has also been reported that VACV infection enhances membrane trafficking between endosomes and the Golgi compartment [52,53,55,99,100]. In addition, the phospholipid composition of the WV membranes is similar to that of the TGN [54]. In support of a role for Golgi or a post-Golgi compartment in wrapping, Rab1a, a protein essential for structural maintenance of endoplasmic reticulum to Golgi transport, was shown to be required for MV wrapping, although no direct interaction of Rab1a with VACV was defined [101].

However, evidence for the involvement of endosomes in WV formation also exists. Using EM in conjunction with fluid phase tracers it was demonstrated that the MV wrapping membranes were likely derived from early endosomes (EEs) [55,56]. Furthermore, interference with retrieval of EV proteins from the plasma membrane via clathrin-mediated endocytosis results in a quantitative reduction in EV yield and delayed virus spread, although no qualitative difference in WV formation was reported [102].

To date, only three viral proteins have been shown to be required for the formation of WVs. The MV associated protein A27, and the two EV specific proteins B5 and F13. Deletion of any one of these from VACV severely inhibits WV formation without impacting the formation of MVs [87,94,103,104,105]. While the A27 protein appears to be important for transport of MVs to the site of wrapping, and B5 for the wrapping process itself, little more information regarding their role in this process is available.

On the other hand several important features of F13, critical for its function in wrapping, have been elucidated [104]. F13 is a non-glycosylated protein, which associates with both of the WV membranes through pamitylation of cysteines 185 and 186 [53,106]. It is located on the cytosolic side of the outermost WV membrane and on the MV-facing side of the inner WV membrane [107]. F13 carries a putative phospholipase D domain (HKD) [108] that is required for its wrapping activity [108]. Interestingly, F13 has been reported to have broad spectrum lipase activity which is thought to mediate Golgi vesicle budding and formation of late endosomes (LEs) containing the various WV proteins [109,110].

In support of this, expression of F13 is required to drive localisation of the other WV proteins, B5 and A36, to LEs [99,100]. In the absence of F13, or upon mutation of its phospholipase D domain these proteins remain in the TGN [99,100]. As no direct interaction between F13 and these proteins has been identified, their LE relocalisation is likely driven by F13’s Golgi budding activity. Importantly, expression of phospholipase D does not rescue EV formation in the absence of F13, implying that F13 has additional roles in MV wrapping, beyond driving vesicle budding [99].

In addition to the phospholipase D domain, F13 contains a conserved tyrosine-tryptophan motif that has been shown to be required for interaction with tail interacting protein of 47 kDa (TIP47) [111]. Mutation of the F13 tyrosine-tryptophan motif results in loss of interaction with TIP47 and abrogation of plaque formation [111]. This late endosomal-derived transport vesicle effector protein interacts with Rab9, a small Ras GTPase, which is also enriched in LEs [112]. Together these proteins mediate receptor recycling from LEs to the TGN [112,113]. Interestingly, Rab9/TIP47 function has also been shown be important for human immunodeficiency virus (HIV), Ebola, Marburg, measles, and hepatitis C virus replication and release, suggesting that the cellular trafficking pathway controlled by these proteins is commonly exploited by enveloped viruses [114,115,116].

Finally, F13 contains a viral late assembly domain (L domain) [117]. These domains, consisting of four-residue motifs, have been identified within many different enveloped virus proteins, and are often important for virus assembly and egress (recently reviewed in [118]). The L domain motif of F13, YPPL, is conserved throughout all orthopoxviruses, and the variant YXXL conserved throughout the *Poxviridae* family [117]. This high level of conservation indicates the importance this domain plays in viral replication. Mutation of the conserved Y and L within this motif results in a virus with a small plaque phenotype, indicative of a defect in virus spread [117].

Interestingly, all viral late domains identified to date interact with members of the endosomal sorting complex required for transport (ESCRT) or one of its associated proteins [119]. ESCRT is a network of cytoplasmic protein complexes (ESCRT-0, ESCRT-I, ESCRT-II, ESCRT-III, Vps4 complex), required for sorting and degradation of ubiquitinated LE membrane proteins.

Briefly, ESCRT-0 recognises ubiquitinated cargo proteins and sequesters them into distinct regions of the LE membrane. Then ESCRT-I/ESCRT-II drive membrane deformation to form buds directed into the lumen of the LE. ESCRT-III is then recruited by ESCRT-I, via ESCRT-II or the accessory protein Alix [120]. Upon its arrival ESCRT-III drives invagination and subsequent membrane fission either on its own, or through interaction with the AAA+ ATPase complex, Vps4. The vesicles formed by this process are released into the lumen of the late endosome, which then becomes known as a multi-vesicular body (MVB). Finally, MVB fusion with lysosomes leads to degradation of the intraluminal vesicles and their associated cargo [121].

ESCRT proteins are also required for numerous other cell trafficking events, such as exosome formation [119]. Exosomes are a type of extracellular vesicle, which when released from cells carry signalling proteins, RNA and lipids to neighbouring cells [121]. Exosomes are formed when the limiting membrane of an MVB fuses with the plasma membrane leading to the release of the intraluminal vesicles into the extracellular space [121]. Unlike canonical intraluminal vesicle formation, sorting of cargo proteins into exosomes does not always depend on ubiquitination of the cargo. It can be driven by direct interaction of the cargo protein with a member of ESCRT or with one of its associated proteins such as Alix [120]. For example, the cytoplasmic protein syntenin interacts directly with Alix to facilitate its packaging into intraluminal vesicles and its eventual exosome-mediated release [122,123].

### 5.3. Is EV Formation an Exosome-Like Process?

**Figure 4:**
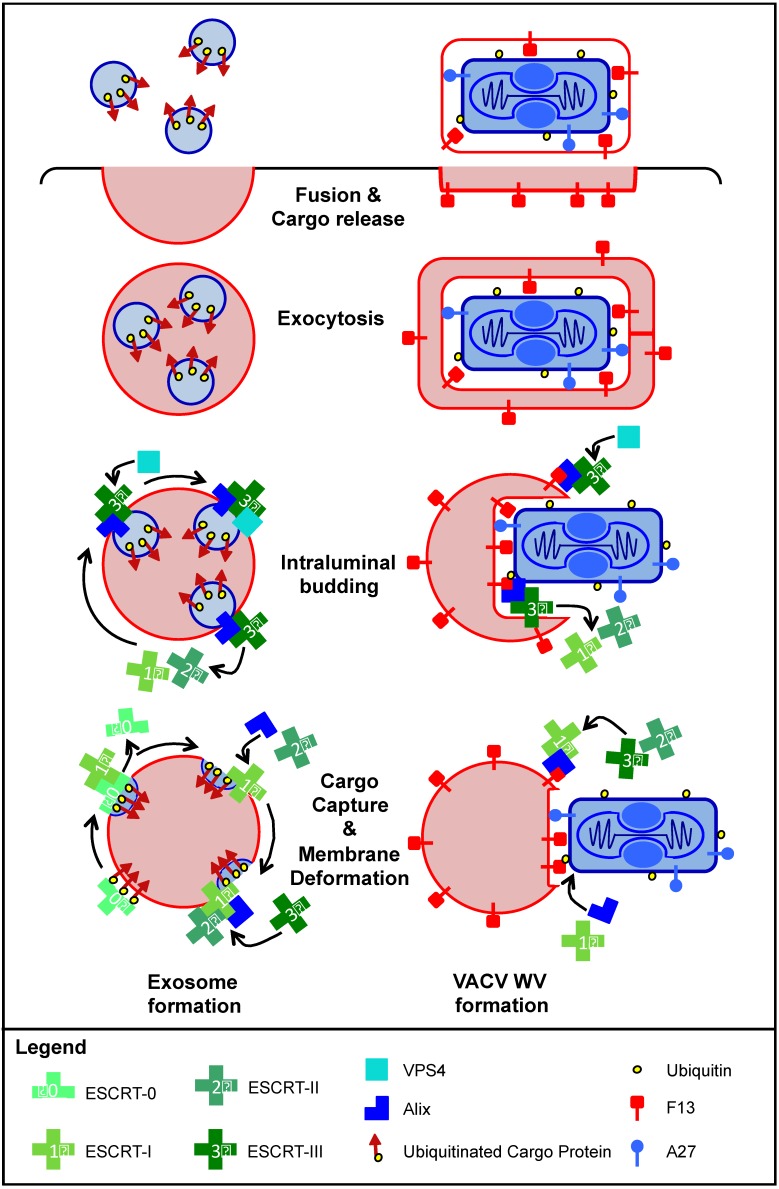
Model for exosome-like extracellular virus (EV) formation. The process of vaccinia virus (VACV) EV formation is highly reminiscent of cellular exosome formation. Both processes proceed through four major steps: Cargo capture and membrane deformation, intraluminal budding, exocytosis and finally fusion with the plasma membrane to release the membrane bound cargo. Canonical exosome formation (left) is regulated by the ESCRTs. ESCRT-0 acts to recognise membrane-bound ubiquitinated cargo proteins and direct them into distinct late endosome (LE) membrane regions. ESCRT-I/ESCRT-II drive membrane deformation. After recruitment of ESCRT-III via ESCRT-II or the accessory protein Alix, ESCRT-I/ESCRT-II depart, and ESCRT-III drives invagination and subsequent membrane fission with assistance of the AAA+ ATPase complex, Vps4. The newly formed multivesicular body (MVB) is transported to the cell surface on microtubules and the intralumenal vesicles are released from the cell when the limiting membrane of the MVB fuses with the plasma membrane, thereby forming exosomes. Based on the evidence described in the text, we propose a model of VACV wrapped virion (WV) formation akin to exosome formation (right). As the EV protein F13 is essential for wrapping, contains a late domain, is present in LEs during infection, and interacts with late endosomal factors, we suggest that F13 acts as an ESCRT-0 mimic that serves to recognise mature viruses (MVs) as cargo for wrapping. While it is unknown what F13 recognises on the MV; both A27, an MV membrane protein required for EV formation, and ubiquitin on the VACV membrane could serve as F13 recognition targets. As an ESCRT-0 mimic, F13 could also serve to recruit ESCRT-I/II and/or ESCRT-III via the accessory protein Alix. This would initiate wrapping, a process topologically analogous to intralumenal budding during MVB formation. In support of this both the ESCRT-1 component, TSG101, and the accessory protein Alix are required for EV formation. To complete WV formation, the Vps4 complex could be recruited to facilitate the sealing of the protective EV membrane. Like exosome release, fully formed WVs require microtubules for transport to the plasma membrane where they fuse, releasing the membrane-bound MV cargo, thus forming the double-membrane EV.

Several viruses have also been shown to hijack exosome formation to mediate their own envelopment and release from host cells. This process was first identified and is best characterised for HIV (reviewed in [119]). Several lines of evidence suggest that VACV may also use an exosome-like pathway to facilitate WV formation (illustrated in Figure 4).

As described above F13 carries an YXXL late domain motif, which when present in viral proteins, are known to interact with the ESCRT accessory protein Alix [117,124,125]. For VACV, depletion of Alix as well as the ESCRT-I component TSG101 has been shown to inhibit EV production [117]. Although no direct interaction between F13 and TSG101 or Alix has been demonstrated, TSG101 is known to interact with Alix [120]. Given that exosome formation via syndecan-syntenin-Alix is known to depend on TSG101 [122], collectively these studies suggest that VACV wrapping may proceed by a similar mechanism.

For recognition as cargo, MVs have been shown to carry membrane associated lipid-modified ubiquitin [126]. As ESCRT-0 initiates exosome formation through recognition of ubiquitinated proteins in LE membranes, perhaps F13 acts as an ESCRT-0 mimic, binding the ubiquitinated MV membrane, recruiting Alix and targeting the MV for exosome-like wrapping. Alternatively, as A27 is the only MV membrane protein essential for EV formation, in addition to transporting MVs to the site of wrapping, maybe A27 also targets MVs for wrapping through direct interaction with F13 or with a cellular factor required for this process [86,103].

Such a model of MV wrapping would dictate that the wrapping membranes are derived from LEs. This is supported by the confocal studies suggesting the F13 mediates transport of B5 and A36 from the TGN to LEs [99,100], and that in the absence of over expression, no Golgi derived proteins are found in EV membranes [127].

Furthermore, the exocytotic release of WVs is highly reminiscent of exosome release mediated by fusion of the outermost MVB membrane with the plasma membrane. Like the fused MVB membrane, the deposited outermost WV membrane is recycled via endocytosis [110,128]. Additional MVs could then be wrapped by EEs now containing the proteins required for WV formation, F13 and B5 [128]. Tip47 interaction with F13 in LEs then mediates recycling of the proteins to the TGN and ensures that B5 and F13 are not trafficked to the lysosome for degradation [111].

## 6. Immune Evasion Role of the EV Membrane

Poxviruses are the only viruses that make a two infectious virus forms. That all poxviruses make EVs and dedicate nine genes to EV formation highlights their importance in the virus lifecycle. The formation of two infectious forms is a clever tactic adopted by VACV. As MVs and EVs display a unique set of membrane proteins VACV forces the host immune system to generate a response to two immunologically distinct invaders. VACV EVs are specifically designed for the purpose of spread. With such a specialised role during infection, not surprisingly the EV membrane provides a number of advantages that help the virus evade immune detection when in the extracellular environment.

Antibodies play a critical protective role against poxvirus infection in humans and primates [129,130]. *In vivo,* EVs are the major form of virus found in circulation, thus their membrane proteins would be predicted to be major targets for protective antibodies generated by infected hosts. Consistent with this notion, EV specific antibodies protect mice and rabbits against lethal challenge better than MV specific antibodies [131,132]. In humans, poxvirus immunisation elicits neutralising antibodies targeted to several MV membrane proteins including A27, L1, H3, and D8, but only B5 on EVs [133,134]. This would suggest that the outer EV membrane acts as a cloak that hides the highly antigenic MV membrane proteins from exposure to the immune system while the virus is in circulation.

In addition, EVs appear to protect their own surface proteins from stimulating humoral immunity. How they achieve this is currently unknown. It seems significant that all membrane proteins displayed on the EV surface are glycosylated while there are no glycosylated membrane proteins packaged into MVs [135,136]. Interestingly, glycosylation of the surface proteins of several viruses including HIV, hepatitis C and gammaherpesvirus, has been shown to shield them from neutralising antibodies [137,138,139]. Although the anti-B5 and the non-neutralising anti-A33 antibodies raised by the human immune response are independent of their glycosylation state [133,134], perhaps EVs employ a glyco-shielding strategy in an attempt to hide their other outer membrane proteins from the humoral immune response.

Several studies to elucidate how antibodies directed against EV proteins mediate protection have lead to the conclusion that complement activity is very important for their protective capacity [140,141]. Both anti-A33 and anti-B5 antibodies combat VACV *in vitro* via complement-mediated virolysis. At high concentrations, anti-B5 can also participate in complement dependent virus opsonisation or trigger complement-mediated lysis of infected host cells [141]. These specific activities have been shown to be important for anti-B5 mediated protection *in vivo* [140,141].

To combat these complement-mediated immune responses VACV has developed a couple of divergent strategies. When deposited on the host cell surface, the EV membrane protein A56 has been shown to bind a virally-encoded complement control protein, C3. In order to block complement-mediated host cell lysis activity VACV C3 binds host complement proteins C3a and C3b thereby abrogating their activity [142]. Although not tested, it is possible that A56 located in the EV membrane administers a similar immune evasion strategy through binding of VACV C3 to EVs to prevent their complement-mediated destruction. The virus also hijacks the host cell proteins CD46, CD55, CD59, CD71, CD81, and major histocompatibility complex class I [84]. Both CD55 and CD59 are complement control proteins that have been shown inhibit the complement-mediated immune response against VACV, both in the presence and absence of EV specific antibodies [84,140]. Thus the EV membrane helps the extracellular virions evade immune detection while in circulation by cloaking the underlying MV, by displaying very few neutralising antibody targets, and through incorporation of host and potentially virus encoded complement control proteins [84,143].

## 7. Perspectives

Likely owing to their large size, exclusively cytoplasmic replication, and large coding capacity, poxviruses have evolved several unique immune evasion strategies that cover the whole of the virus lifecycle from entry to spread. While apoptotic mimicry has been linked to VACV entry it is an attractive possibility that apoptotic mimicry may also facilitate engagement of PS receptors to dampen the host immune response. This strategy would provide VACV with the possibility to modulate cellular immunity prior to entering cells and without the need to encode and package additional viral proteins. Furthermore, the broad cell type and tissue tropism of VACV may be attributable to the existence of multiple PS-receptors and the ability of both professional and non-professional phagocytes to clear apoptotic debris [144,145]. A detailed analysis of the host signalling pathways activated by VACV will be important for understanding the multifaceted role of PS receptors in binding, endocytosis, infection, and innate immune suppression during infection of relevant cell types and *in vivo*. Perhaps most importantly, the therapeutic potential of targeting viral PS to prevent poxvirus infection should be investigated [146].

In addition to expressing a large subset of immunomodulatory proteins [18], poxviruses uniquely carry LBs that allow for the delivery of potential immunomodulatory factors prior to gene expression. The advantages of such a strategy range from shunting of antiviral innate immune responses to establishing a favorable cytoplasmic environment for DNA replication prior to genome release. Interestingly another large DNA virus, herpes simplex virus 1 (HSV1), also carries a proteinaceous layer between its capsid and envelope, termed tegument [14]. Akin to poxviruses LBs, upon entry some HSV1 tegument proteins are shed into the cytoplasm in order to modulate host cell activities [14]. The tegument protein pUL13 for example has been shown to inhibit the type I IFN response, while pUL41 and pUL34.5 down regulate expression of major histocompatibility complex class II [16,17].

To date only three LB components have been identified [15]. By analogy to HSV1 tegument, it is tempting to hypothesise that the remaining mass of poxvirus LBs account for an artillery of viral encoded immune modulatory enzymes that serve to shut down very early cellular immune responses to infection. Identification and characterisation of the remaining LB constituents deserves further work. Such studies may serve to confirm an immune-modulating function of LBs and identify the early immune pathways that sufficiently threaten invading *Poxviridae* such that these viruses have evolved to defuse them.

Poxviruses have, at least in part, evolved EVs to allow the virus to spread faster than it replicates, via superinfection repulsion [51]. As EVs are the first form of virus released into the extracellular environment after initial infection, it reasons that they are provided with an additional cloak to protect them from host immune detection and destruction. EVs achieve this by masking the underlying MV, potentially glyco-shielding EV proteins from antibody recognition, and by packaging viral and host proteins to block complement mediated destruction. While much is known about the individual viral proteins required for EV formation [57,135], several interesting aspects of this process await further investigation. These include the microtubule motors that transport MVs to the site of wrapping, the viral and cell factors involved in WV fusion at the plasma membrane, and as discussed in this review the cellular membrane source, mechanism, and cell factors that facilitate WV formation.

Collectively, these unique immune evasion strategies are likely to provide researchers the opportunity to define novel immunomodulatory functions of poxviruses and in turn the possibility of uncovering previously undefined cellular innate and intrinsic immune responses to viral infection. Furthermore, in depth understanding of how poxviruses modulate the immune system is likely to lead to better antiviral therapeutic design and smarter oncolytic poxvirus development [147,148].
